# Retracted: Role of Wheat Based Diet on the Pathology of Necrotic Enteritis in Turkeys

**DOI:** 10.1155/2018/6946408

**Published:** 2018-06-06

**Authors:** Scientifica

At the request of the authors, the article titled “Role of Wheat Based Diet on the Pathology of Necrotic Enteritis in Turkeys” [1] has been retracted. The article was found to contain a substantial amount of material from published sources. The first author confirmed that all the experiments were carried out in Pakistan, and the affiliation to the National Veterinary School of Toulouse, France, was added by mistake. Muhammad Younus and Muhammad Ali Abdullah Shah did not contribute to this research and should not be considered as coauthors of this article. They were added by Dr. Umar and the journal in error, without their knowledge or permission. The journal apologizes for the mistake of not confirming their authorship.
